# Diagnostic performance of Vcheck NT-proBNP and cardiac troponin I assays for detection of dilated cardiomyopathy in Doberman Pinschers

**DOI:** 10.3389/fvets.2026.1895815

**Published:** 2026-09-14

**Authors:** Helena Driessen, Jana Friederich, Gerhard Wess

**Affiliations:** LMU Small Animal Clinic, Centre for Clinical Veterinary Medicine, Faculty of Veterinary Medicine, Ludwig-Maximilians-University, Munich, Germany

**Keywords:** cardiac biomarkers, early disease detection, Holter monitoring, point-of-care testing, screening

## Abstract

**Background:**

Dilated cardiomyopathy (DCM) is a common hereditary cardiac disease in Doberman Pinschers. Early myocardial changes can be detected using cardiac biomarkers such as N-terminal pro-B-type natriuretic peptide (NT-proBNP) and cardiac troponin I (cTnI). The Vcheck V200 (Bionote) is a point-of-care immunofluorescence-based assay for in-clinic measurement of these markers.

**Objective:**

To evaluate the diagnostic performance of NT-proBNP and cTnI measured with the Vcheck V200 point-of-care assays for detecting different stages of DCM in Doberman Pinschers and to compare Vcheck cTnI results with those obtained using a laboratory-based IDEXX cTnI assay.

**Animals:**

A total of 453 serum samples from 345 privately owned, purebred Doberman Pinschers in Germany were analyzed. Dogs were clinically classified into five stages (A, last normal, B1, B2, and C) based on echocardiographic and electrocardiographic findings.

**Methods:**

This prospective longitudinal study included physical examination, echocardiography, 24-h Holter monitoring, and biomarker analysis. NT-proBNP and cTnI concentrations were measured using the Vcheck V200 assay. In a subset of 358 samples, cTnI concentrations were additionally measured using the IDEXX assay. Diagnostic performance was evaluated using receiver operating characteristic curve analysis, and agreement between cTnI assays was assessed using Bland–Altman analysis.

**Results:**

NT-proBNP and cTnI concentrations were significantly higher in dogs with DCM than in healthy controls. For NT-proBNP measured with Vcheck, a cutoff of ≥580 pmol/L yielded 76.6% sensitivity and 83.1% specificity (AUC 0.845). For cTnI measured with Vcheck, a cutoff of ≥0.21 ng/mL achieved 71.0% sensitivity and 78.3% specificity (AUC 0.809). The IDEXX cTnI assay showed higher diagnostic accuracy, particularly in early-stage disease, including “last normal” dogs. Combining both Vcheck biomarkers increased sensitivity and overall diagnostic performance (AUC 0.886), but specificity was reduced and performance remained lower than that of IDEXX cTnI alone.

**Conclusion and clinical importance:**

The Vcheck V200 provides a practical and accessible tool for in-clinic biomarker assessment in Doberman Pinschers at risk for DCM. While Vcheck NT-proBNP and cTnI may help identify dogs requiring further cardiac evaluation, the laboratory-based IDEXX cTnI assay showed higher diagnostic accuracy, especially for early-stage detection. Dogs exceeding these biomarker thresholds should undergo comprehensive cardiac evaluation, including echocardiography and 24-h Holter monitoring.

## Introduction

Dilated cardiomyopathy (DCM) is an inherited cardiac disease in Doberman Pinschers (1), with a reported prevalence of 58% in Europe (2). The disease is characterized by a prolonged occult stage, often lasting several years, during which affected dogs remain clinically asymptomatic despite the presence of echocardiographic and/or electrocardiographic abnormalities (3, 4). Echocardiographic findings typically include left ventricular systolic and diastolic enlargement (5), whereas electrocardiographic abnormalities most commonly manifest as ventricular premature complexes (VPCs) (4). These structural and electrical abnormalities may occur independently or concurrently and can progress to ventricular tachycardia, ventricular fibrillation, sudden cardiac death, or congestive heart failure (3, 6).

The occult stage of DCM is most commonly diagnosed between 5 and 7 years of age, although affected dogs may be identified as early as 2 years of age (3, 7, 8). The occult stage of DCM may manifest as isolated ventricular arrhythmias despite normal cardiac dimensions (stage B1) or as echocardiographic evidence of cardiac enlargement with or without arrhythmias in the absence of clinical signs of congestive heart failure (stage B2), before progressing to overt disease (stage C) (9).

Disease progression is typically gradual during the occult phase but may accelerate once clinical signs develop (4). Although no overall sex predisposition has been demonstrated, phenotypic differences have been reported between sexes. Female Doberman Pinschers more frequently exhibit ventricular arrhythmias in the absence of echocardiographic abnormalities, whereas males tend to develop structural cardiac changes at an earlier stage of the disease (2, 3).

Early identification of occult DCM is important for both clinical management and breeding decisions. Detecting affected dogs before the onset of clinical signs may facilitate timely diagnostic follow-up and therapeutic intervention, while also supporting breeding programs aimed at reducing the transmission of disease-associated traits. Because echocardiography and 24-h Holter monitoring require specialized equipment and expertise that may not be readily available in all veterinary practices, accessible screening tools are desirable (3, 9). One promising approach is the use of circulating cardiac biomarkers, which have been investigated as adjunctive tools for identifying Doberman Pinschers at risk for DCM (10–12).

Recently, the Vcheck V200 (Bionote Inc., Hwaseong-si, South Korea), a point-of-care immunofluorescence-based analyzer, became available for in-clinic quantitative measurement of NT-proBNP and cardiac troponin I (cTnI). This platform enables rapid biomarker assessment and may provide a practical alternative when advanced cardiac diagnostics are not immediately available. Analytical validation studies have shown that the Vcheck NT-proBNP assay fulfills ASVCP/CLIA performance criteria and demonstrates excellent agreement with reference laboratory measurements (13). NT-proBNP is released in response to increased myocardial wall stress (14). Previous studies in Doberman Pinschers demonstrated significantly higher NT-proBNP concentrations in dogs with DCM compared to healthy controls. A cutoff value of >550 pmol/L identified dogs with echocardiographic evidence of DCM with a sensitivity of 78.6% and a specificity of 90.4%, whereas lowering the threshold to >400 pmol/L increased sensitivity to 90% at the expense of specificity (75%) (11). More recently, Dukes-McEwan et al. reported that NT-proBNP screening was useful for identifying Doberman Pinschers with occult DCM and proposed a cutoff value of >626 pmol/L with a sensitivity of 79% and a specificity of 81% (12). However, NT-proBNP appears to have limited ability to identify dogs with isolated arrhythmias and no structural cardiac abnormalities (stage B1) (11).

Cardiac troponin I is a structural protein of the cardiac sarcomere and is released into the circulation following myocardial injury. Previous studies have shown that cTnI concentrations are increased in Doberman Pinschers with DCM across different stages of disease (15). Using a first-generation assay, a cutoff value of >0.22 ng/mL yielded a sensitivity of 79.5% and a specificity of 84.4% for the detection of DCM (15). More recently, a high-sensitivity cTnI assay demonstrated improved detection of early disease stages, and concentrations >0.113 ng/mL were proposed as a screening threshold warranting further cardiologic evaluation, including echocardiography and Holter monitoring (16). Although cardiac biomarkers do not replace echocardiographic and electrocardiographic examination, they may provide valuable support for identifying dogs that require comprehensive cardiac assessment (3). Although the diagnostic relevance of NT-proBNP and cTnI in Doberman Pinschers has previously been established, the clinical diagnostic performance of these biomarkers depends on the specific analytical platform used. The present study therefore focused on the clinical performance of the Vcheck V200 point-of-care assays rather than on re-establishing the biological relevance of the biomarkers themselves.

The primary aim of this study was to evaluate the clinical diagnostic performance of NT-proBNP and cTnI measured with the Vcheck V200 point-of-care assay for detecting different stages of DCM in Doberman Pinschers and to determine clinically applicable assay-specific screening thresholds. A secondary aim was to compare the diagnostic performance and agreement of Vcheck cTnI measurements with those obtained using a laboratory-based high-sensitivity IDEXX cTnI assay. We hypothesized that the Vcheck assays would provide clinically useful diagnostic information, but that the high-sensitivity laboratory assay would demonstrate superior performance, particularly for the detection of very early disease.

## Materials and methods

The study included 453 serum samples obtained from 345 privately owned, purebred Doberman Pinschers in Germany. Samples were collected as part of an ongoing prospective longitudinal screening program, and some dogs contributed samples from multiple examinations over time. Each serum sample was analyzed for NT-proBNP and cardiac troponin I (cTnI) using the Vcheck V200 assay.

Only Doberman Pinschers without evidence of relevant systemic disease were included. Dogs underwent clinical evaluation at least annually, including collection of medical history, physical examination, echocardiography, a short electrocardiogram (ECG), and 24-h Holter monitoring. Healthy dogs were re-evaluated annually, whereas dogs diagnosed with DCM were typically re-examined every 3 months.

Echocardiographic examinations were performed without sedation by a board-certified cardiologist or a cardiology resident under direct supervision, with dogs positioned in right and left lateral recumbency. The Simpson method of discs (SMOD) was used as the primary echocardiographic method for classification of Doberman cardiomyopathy and was performed from the right parasternal long-axis view and the left apical four-chamber view. Dogs were classified as having echocardiographic evidence of DCM if the left ventricular end-systolic volume indexed to body surface area (LVESV/BSA) exceeded 55 mL/m^2^, the left ventricular end-diastolic volume indexed to body surface area (LVEDV/BSA) exceeded 95 mL/m^2^, or both measurements exceeded these thresholds (3).

M-mode measurements obtained from the right parasternal long-axis view were used as supportive parameters only and were not used as the primary basis for disease classification. Reference values for M-mode measurements were considered exceeded when LVIDs was >3.6 cm and LVIDd was >4.6 cm in females or >4.8 cm in males (3). Valvular insufficiencies were assessed using color Doppler echocardiography. Continuous-wave Doppler measurements across the aortic and pulmonary valves were required to remain within physiological limits.

24-h Holter monitoring was performed at each examination and analyzed using Amedtech (Medizintechnik Aue GmbH, Aue-Bad Schlema, Germany). All recordings were reviewed by a trained cardiologist, who verified arrhythmias and manually corrected VPC detections when necessary.

Dogs were assigned to clinical stages based on echocardiographic findings obtained by SMOD and 24-h Holter monitoring according to established screening criteria for Doberman cardiomyopathy:

1. Stage A: No clinical signs, <50 VPCs/24 h, normal echocardiographic measurements.
2. Last-normal: Dogs with normal echocardiographic and Holter monitoring findings at the time of sample collection that developed DCM within 12 months, as confirmed by standard screening procedures including echocardiography (SMOD) and 24-h Holter monitoring.
3. Stage B1: No clinical signs, normal echocardiographic measurements, but >50 VPCs/24 h on at least two separate occasions.
4. Stage B2: Abnormal echocardiographic measurements with or without VPCs, but no clinical symptoms.
5. Stage C: Overt disease with clinical symptoms (e.g., syncope, exercise intolerance, cough, or dyspnea due to congestive heart failure) and abnormal echocardiographic and ECG findings.

Dogs were excluded if echocardiographic findings were considered equivocal (i.e., cardiac dimensions were partially within and partially outside the established reference ranges) or if assignment to one of the five predefined clinical stages was not possible. Dogs with relevant systemic diseases or with congenital or acquired cardiac diseases other than Doberman cardiomyopathy were also excluded. Blood samples were collected by venipuncture into serum tubes, centrifuged after collection, and stored at −80 °C until analysis. The maximum storage duration was 9 years and 6 months. For Vcheck measurements, frozen serum samples were thawed at room temperature for approximately 30 min prior to analysis. NT-proBNP and cTnI concentrations were measured using the Vcheck V200 analyzer according to the manufacturer’s instructions. For comparison of cTnI measurements, results obtained with a laboratory-based high-sensitivity cTnI assay (IDEXX Laboratories) were available for a subset of dogs as part of routine clinical screening. These results were retrospectively retrieved and used for comparison with the corresponding Vcheck cTnI measurements. Vcheck measurements were performed without knowledge of the dogs’ clinical stage or the corresponding IDEXX cTnI results.

### Statistical analysis

Statistical analyses were performed using IBM SPSS Statistics (Version 28.01.00; IBM Corp., Armonk, NY, USA) and MedCalc Statistical Software (Version 23.2.1; MedCalc Software Ltd., Ostend, Belgium) for selected analyses. A significance level of *p* < 0.05 was considered statistically significant. Descriptive statistics, including mean, standard deviation, median, minimum, and maximum values, were calculated for each clinical stage of Doberman cardiomyopathy. Data distribution was assessed using the Kolmogorov–Smirnov test. Because the study was based on a longitudinal screening program, some dogs contributed more than one sample over time, and observations were therefore not fully independent. The primary analyses were nevertheless performed at the sample level to evaluate biomarker performance across different disease stages. No mixed-effects models, generalized estimating equations, or cluster-adjusted ROC analyses were applied.

To compare biomarker concentrations and demographic characteristics across disease stages, the Kruskal–Wallis test was applied, followed by Mann–Whitney U tests for pairwise comparisons. When appropriate, *p*-values were adjusted using the Bonferroni correction to account for multiple comparisons. The influence of age and sex on biomarker concentrations in the healthy control group (stage A) was analyzed using linear regression analysis. To assess the potential impact of sample storage duration on biomarker concentrations, linear regression analyses were performed in the entire study population.

Receiver operating characteristic (ROC) curve analyses were performed to evaluate the diagnostic performance of NT-proBNP and cTnI (Vcheck and IDEXX). Areas under the curve (AUCs), sensitivity, specificity, and optimal cutoff values based on the Youden index were calculated for each ROC analysis. For clinical interpretation and comparison with previously published studies, selected cutoff values of 580 pmol/L for NT-proBNP (Vcheck), 0.21 ng/mL for cTnI (Vcheck), and 0.11 ng/mL for cTnI (IDEXX) were used for the primary analyses presented in this study. AUC comparisons between biomarkers and measurement platforms were performed using the nonparametric DeLong test in MedCalc.

Agreement between Vcheck and IDEXX cTnI measurements was assessed using Bland–Altman analysis. One extreme outlier with a mean cTnI concentration exceeding 6 ng/mL was excluded because of its disproportionate influence on the limits of agreement. The mean difference (bias) and the 95% limits of agreement were subsequently calculated and visualized.

To evaluate whether combining NT-proBNP and cTnI (both measured with the Vcheck platform) improved diagnostic performance over individual biomarkers, a logistic regression model was constructed in SPSS. The resulting model was evaluated using ROC analysis. Comparisons of AUCs between the logistic regression model and individual markers were performed using MedCalc. All statistical tests were two-sided and based on 95% confidence intervals.

## Results

### Group characteristics

A total of 453 serum samples obtained from 345 dogs were included in the analyses. The median age of the study population was 6.4 years (range, 1.0–13.6 years). Dogs in stage A were significantly younger than dogs in all other clinical groups (*p* < 0.001 for all comparisons). In addition, dogs in the B1 group were significantly older than dogs in the LN group (*p* = 0.002), whereas no other age differences were identified. Of the 345 dogs, 186 were male and 159 were female. Sex distribution did not differ significantly among clinical groups (*p* = 0.078), although a higher proportion of males was observed in stages B2 and C (Table 1). For comparison of cTnI measurements between Vcheck and IDEXX, paired results were available from 358 serum samples. The distribution of samples across clinical stages was comparable to that of the overall study population.

**Table 1:** Demographic characteristics and echocardiographic measurements of dogs across clinical stages A to C.

	A	LN	B1	B2	C
Samples	83	80	85	143	62
Female (*n*)	43	38	48	56	25
Male (*n*)	40	42	37	87	37
Age (years), mean ± SD	4.16 ± 1.92	6.16 ± 2.34	7.33 ± 2.39	6.94 ± 2.47	7.19 ± 2.72
LVEDV/BSA, mean ± SD	71.60 ± 10.13	77.99 ± 13.21	72.46 ± 12.34	109.91 ± 22.32	134.52 ± 42.85
LVESV/BSA, mean ± SD	40.18 ± 7.68	43.05 ± 9.52	40.26 ± 9.00	69.22 ± 19.35	100.80 ± 35.21
LA/Ao, mean ± SD	1.30 ± 0.13	1.31 ± 0.11	1.31 ± 0.15	1.39 ± 0.21	1.99 ± 0.46
LVIDd, mean ± SD	4.00 ± 0.28	4.24 ± 0.37	4.13 ± 0.37	4.96 ± 0.56	5.76 ± 0.90
LVIDs, mean ± SD	3.07 ± 0.32	3.31 ± 0.37	3.19 ± 0.37	4.11 ± 0.60	5.02 ± 0.87

Values are presented as mean ± standard deviation (SD). LVEDV/BSA and LVESV/BSA refer to left ventricular end-diastolic and end-systolic Simpson volumes indexed to body surface area. LA/Ao = left atrial to aortic root ratio; LVIDd = left ventricular internal diameter in diastole; LVIDs = left ventricular internal diameter in systole.

### Evaluation of age, sex, and sample storage duration on Vcheck cardiac biomarker concentrations

All analyses regarding age and sex were performed in the healthy control group (stage A). No significant association was observed between age and either cTnI (*R*^2^ = 0.017, *p* = 0.240) or NT-proBNP concentrations (*R*^2^ = 0.009, *p* = 0.383). A significant effect of sex was identified for NT-proBNP (*R*^2^ = 0.049, *p* = 0.044), with male dogs exhibiting slightly higher concentrations than females. According to the regression coefficient (B = 75.917), NT-proBNP concentrations were approximately 76 pmol/L higher in males. No sex-related difference was observed for cTnI concentrations (*R*^2^ = 0.003, *p* = 0.619).

No significant association was found between sample storage duration and either cTnI (*R*^2^ = 0.000, *p* = 0.849) or NT-proBNP concentrations (*R*^2^ = 0.001, *p* = 0.480).

### Evaluation of NT-proBNP and cTnI for the detection of Doberman cardiomyopathy using Vcheck and IDEXX

Both NT-proBNP and cTnI concentrations were significantly increased in dogs with DCM compared to healthy controls. Detailed diagnostic performance metrics, including sensitivity, specificity, cutoff values, and AUCs, are summarized in Table 2.

**Table 2:** Diagnostic performance of NT-proBNP (Vcheck), cTnI (Vcheck), and cTnI (IDEXX) for detecting different stages of Doberman cardiomyopathy.

Diseased stages vs. healthy stage A	NT-proBNP (Vcheck) cutoff ≥580 pmol/L	cTnI (Vcheck) cutoff ≥0.21 ng/mL	cTnI (IDEXX) cutoff ≥0.11 ng/mL	ΔAUC comparisons (DeLong test)
B1, B2, C	Sens 76.6% Spec 83.1% AUC 0.845	Sens 71.0% Spec 78.3% AUC 0.809	Sens 78.8% Spec 97.0% AUC 0.936	IDEXX vs. Vcheck cTnI: 0.127 (*p* < 0.0001) IDEXX vs. NT-proBNP: 0.091 (*p* = 0.0002) NT-proBNP vs. Vcheck cTnI: 0.036 (ns)
B1	Sens 62.4% Spec 82.5% AUC 0.747	Sens 64.7% Spec 78.3% AUC 0.761	Sens 60.3% Spec 97.6% AUC 0.867	IDEXX vs. Vcheck cTnI: 0.106 (*p* < 0.05) IDEXX vs. NT-proBNP: 0.120 (*p* = 0.0182) NT-proBNP vs. Vcheck cTnI: −0.014 (ns)
B2	Sens 75.5% Spec 83.1% AUC 0.839	Sens 65.0% Spec 78.3% AUC 0.783	Sens 80.0% Spec 96.4% AUC 0.952	IDEXX vs. Vcheck cTnI: 0.169 (*p* < 0.0001) IDEXX vs. NT-proBNP: 0.113 (*p* = 0.0002) NT-proBNP vs. Vcheck cTnI: 0.056 (ns)
LN	Sens 52.5% Spec 82.5% AUC 0.724	Sens 56.9% Spec 78.9% AUC 0.739	Sens 53.9% Spec 97.0% AUC 0.841	IDEXX vs. Vcheck cTnI: 0.102 (*p* = 0.0468) IDEXX vs. NT-proBNP: 0.117 (*p* = 0.0268) NT-proBNP vs. Vcheck cTnI: −0.015 (ns)
ALL	Sens 71.4% Spec 83.1% AUC 0.819	Sens 68.4% Spec 78.3% AUC 0.794	Sens 72.9% Spec 97.0% AUC 0.913	IDEXX vs. Vcheck cTnI: 0.119 (*p* = 0.0001) IDEXX vs. NT-proBNP: 0.094 (*p* = 0.0002) NT-proBNP vs. Vcheck cTnI: 0.025 (ns)

Shown are the cutoff values, sensitivity, specificity, and AUCs determined by ROC analysis for each biomarker across different group comparisons (stages B1, B2, C vs. A; B1 vs. A; B2 vs. A; LN vs. A; and all stages vs. A). All ROC analyses were statistically significant (*p* < 0.001). Comparisons of AUCs between assays were performed using the DeLong test. ΔAUC denotes the difference in AUC values between the two tests (positive values indicate higher AUC for the first-listed assay). Ns = not statistically significant (*p* > 0.05).

Among the Vcheck biomarkers, NT-proBNP showed the highest diagnostic performance in the comparison of dogs with DCM (stages B1, B2, and C) versus healthy controls (AUC 0.845). Diagnostic performance was comparable in dogs with structural cardiac changes (B2 vs. A; AUC 0.839) but lower in dogs with isolated arrhythmias (B1 vs. A; AUC 0.747).

Cardiac troponin I measured with the Vcheck assay showed slightly lower overall diagnostic performance than NT-proBNP, with the highest AUC observed in the comparison of stages B1, B2, and C versus stage A (AUC 0.809). Performance was similar for the detection of isolated arrhythmic disease (B1 vs. A; AUC 0.761).

The laboratory-based IDEXX cTnI assay achieved the highest diagnostic performance across all comparisons. The greatest diagnostic accuracy was observed for the detection of dogs with structural cardiac changes (B2 vs. A; AUC 0.952). In addition, IDEXX cTnI demonstrated the strongest performance in identifying dogs classified as LN, indicating improved detection of very early disease stages (Table 2). The distribution of NT-proBNP and cTnI concentrations across clinical stages is illustrated in Figures 1, 2.

**Figure 1:**
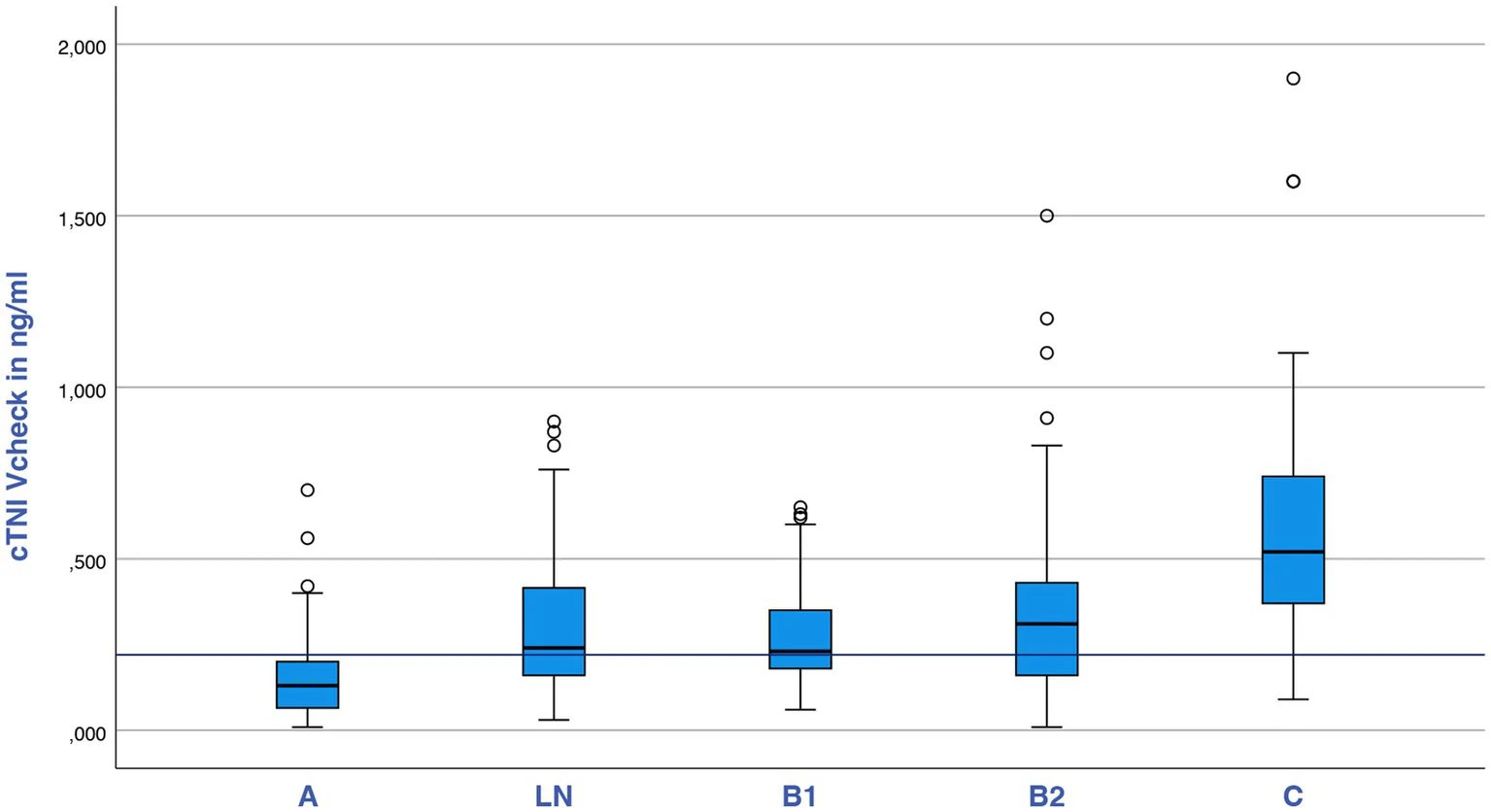
Boxplots of cTnI concentrations measured with the Vcheck assay across clinical stages of Doberman cardiomyopathy. The blue horizontal lines indicate the respective diagnostic cutoffs; outliers are shown as dots.

**Figure 2:**
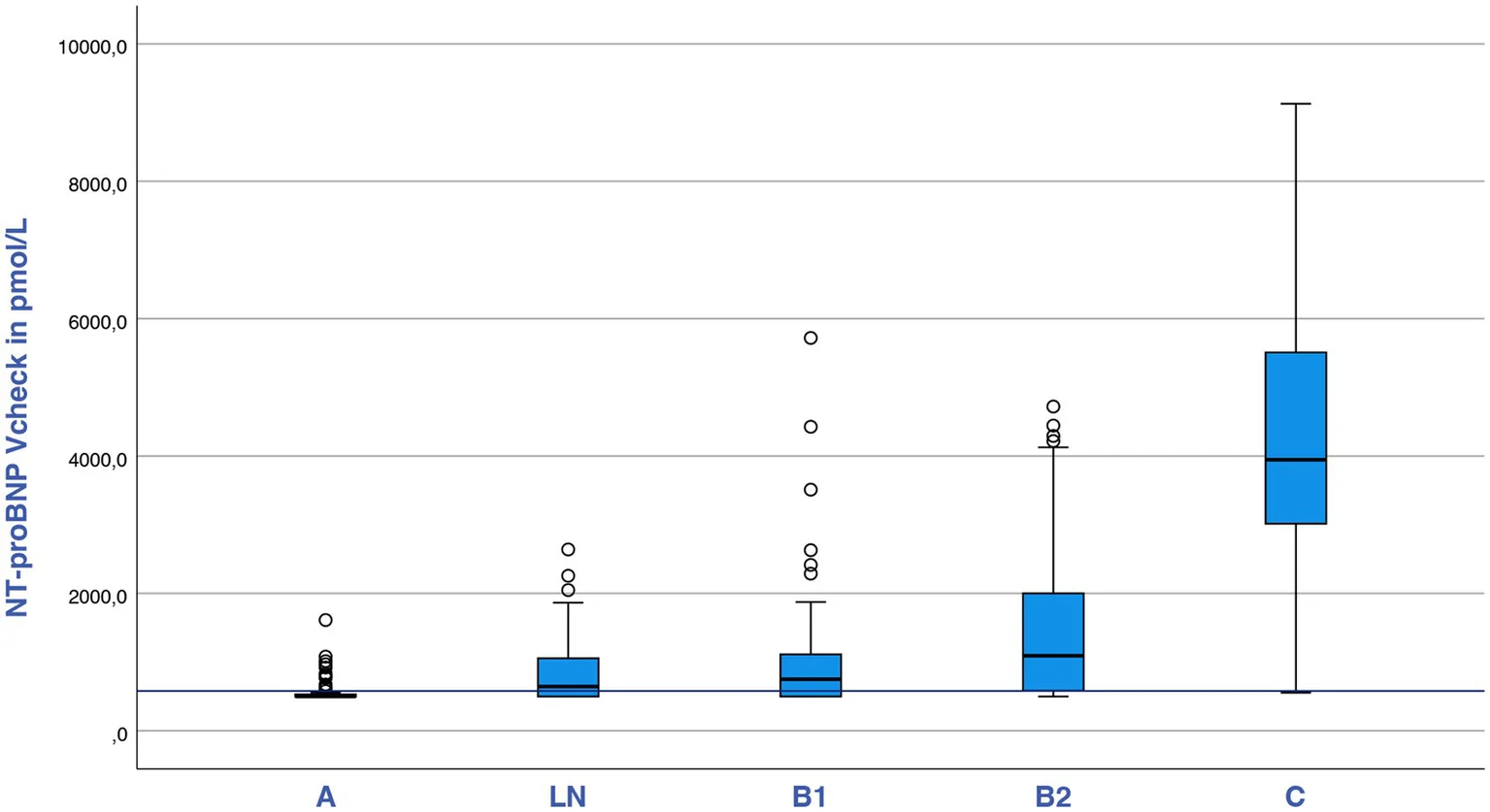
Boxplots of NT-proBNP concentrations measured with the Vcheck assay across clinical stages of Doberman cardiomyopathy. The blue horizontal lines indicate the respective diagnostic cutoffs; outliers are shown as dots.

### Analytical test comparison between the Vcheck cTnI assay and the IDEXX cTnI assay

Agreement between cTnI measurements obtained with the Vcheck and IDEXX assays was evaluated using Bland–Altman analysis of 357 paired samples. The mean difference (bias) between methods was 0.0817 ng/mL, with 95% limits of agreement ranging from −0.361 to 0.525 ng/mL. Vcheck systematically yielded higher cTnI concentrations than IDEXX (*p* < 0.001) (Figure 3). One extreme outlier with a mean cTnI concentration exceeding 6 ng/mL was excluded because of its disproportionate influence on the limits of agreement. The value was considered non-representative of the overall measurement distribution and was therefore omitted from the Bland–Altman analysis.

**Figure 3:**
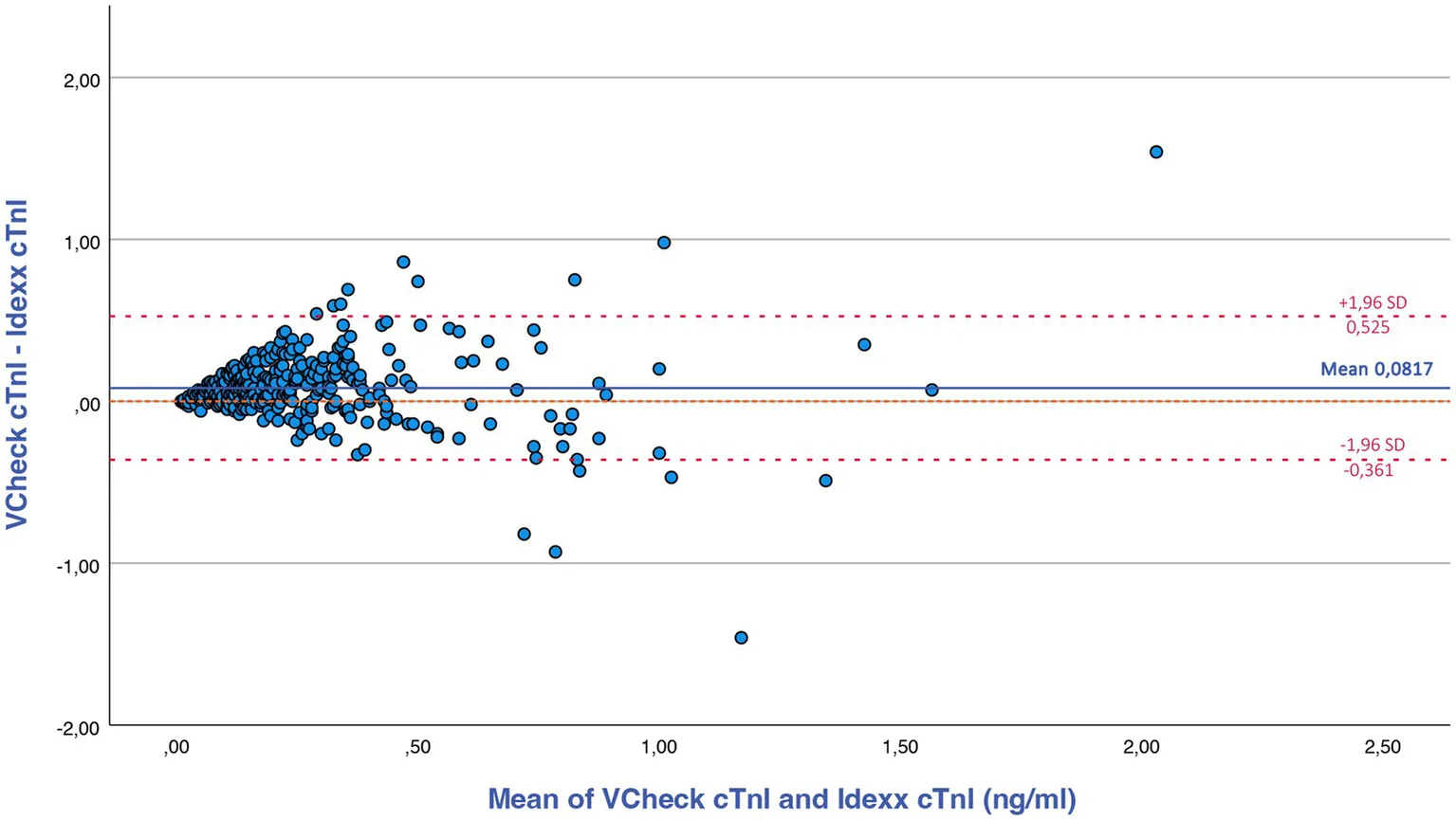
Bland-Altman plot comparing Vcheck and IDEXX measurements of canine cardiac troponin I (cTnI). The plot displays the difference between Vcheck and IDEXX values (y-axis) against their mean (x-axis) for each individual measurement. The solid blue line represents the mean difference (bias = 0.0817 ng/mL), while the dashed red lines indicate the 95% limits of agreement (−0.361 to 0.525 ng/mL).

### Evaluation of inter-assay differences in ROC performance of cardiac biomarkers using Vcheck and IDEXX

Differences in diagnostic performance between biomarkers and assay platforms were assessed by comparing ROC curve AUCs using the DeLong test (Table 2). Overall, cTnI measured with the IDEXX assay achieved significantly higher AUCs than cTnI measured with Vcheck across all group comparisons. IDEXX cTnI also outperformed NT-proBNP measured with Vcheck in all analyses.

In contrast, no significant differences were detected between NT-proBNP and cTnI when both biomarkers were measured using the Vcheck platform. NT-proBNP tended to perform better in dogs with structural cardiac changes (B2 vs. A), whereas Vcheck cTnI showed slightly higher diagnostic accuracy in dogs with isolated arrhythmias (B1 vs. A). Detailed AUC differences and corresponding *p*-values are presented in Table 2. Representative ROC curves illustrating the diagnostic performance of the evaluated biomarkers are shown in Figure 4.

**Figure 4:**
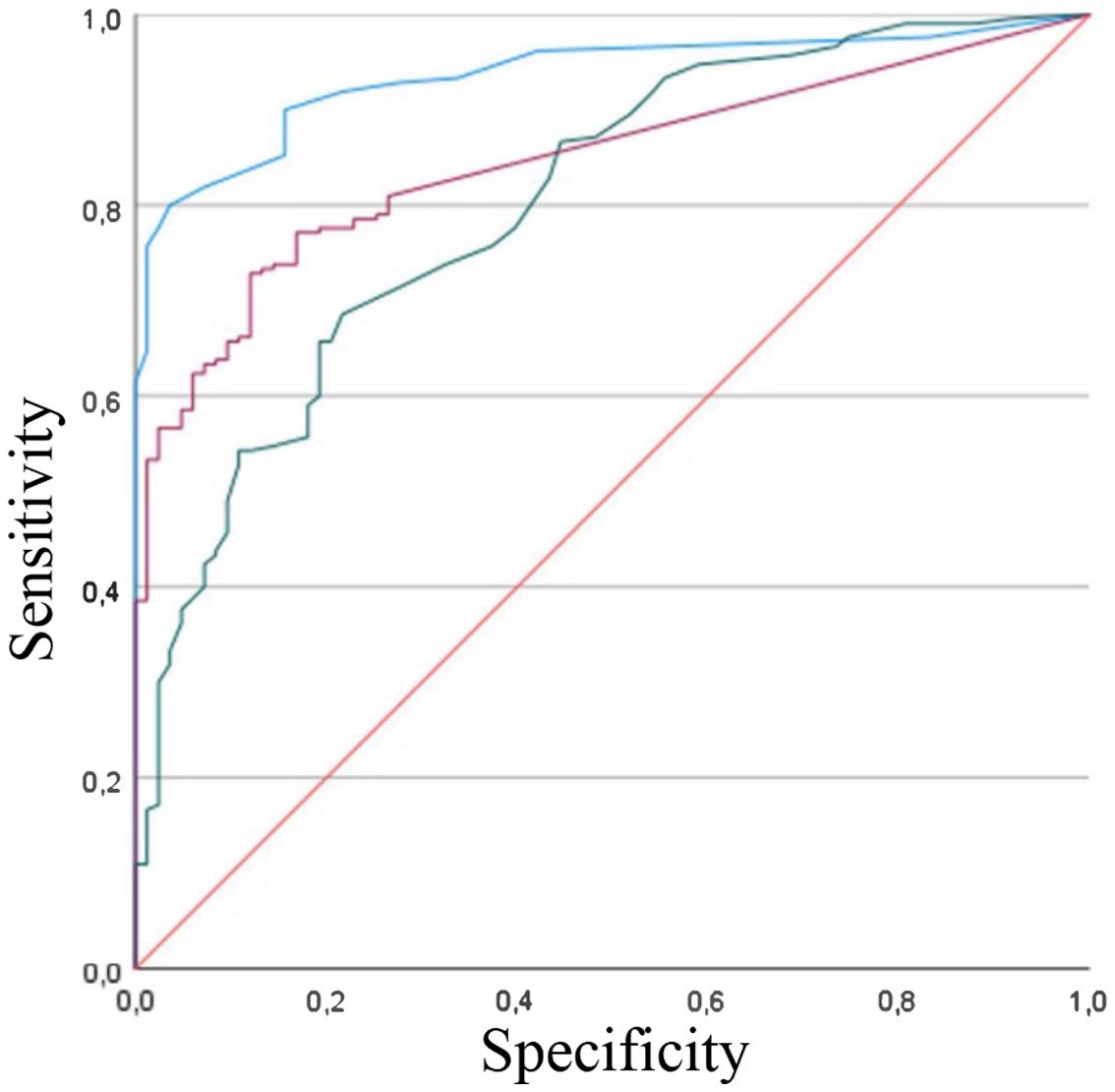
Receiver operating characteristic (ROC) curves illustrating the diagnostic performance of cTnI (IDEXX and Vcheck) and NT-proBNP (Vcheck) for detection of DCM in Doberman Pinschers. ROC curves are shown for cTnI measured using IDEXX (blue), cTnI using Vcheck (green) and NT-proBNP measured using Vcheck (purple). The red line represents the line of no discrimination (AUC = 0.5). AUC reflects the overall diagnostic accuracy of each biomarker.

### Diagnostic utility of combined NT-proBNP and cTnI measurement using Vcheck

The diagnostic utility of combining NT-proBNP and cTnI measured with the Vcheck assay was evaluated using a logistic regression model. In the comparison of dogs with DCM (stages B1, B2, and C) versus healthy controls (stage A), the combined model achieved an AUC of 0.886 (95% CI: 0.851–0.921), compared with 0.845 (95% CI: 0.805–0.885) for NT-proBNP alone and 0.809 (95% CI: 0.758–0.860) for cTnI alone.

At the selected cutoff, the combined model identified 262 of 290 diseased dogs (sensitivity 90.3%), whereas specificity decreased to 55.4%. Compared with NT-proBNP alone at the selected cutoff of ≥580 pmol/L, the combined approach identified 40 additional diseased dogs; compared with Vcheck cTnI alone at the selected cutoff of ≥0.21 ng/mL, it identified 56 additional diseased dogs. The AUC of the combined model was significantly higher than that of cTnI measured with Vcheck alone (ΔAUC = 0.077; *p* = 0.0147), but did not differ significantly from NT-proBNP alone (ΔAUC = 0.041; *p* = 0.1306). However, the combined model remained significantly inferior to cTnI measured with the IDEXX assay (AUC 0.936; ΔAUC = −0.050; *p* = 0.0277).

### Early detection: ROC analysis in the comparison of “last normal” vs. healthy dogs

In the comparison of LN dogs and healthy controls, cTnI measured with the IDEXX assay achieved the highest diagnostic accuracy (AUC 0.841), outperforming both cTnI (Vcheck; AUC 0.739) and NT-proBNP (Vcheck; AUC 0.724). The differences between IDEXX cTnI and both Vcheck-based biomarkers were statistically significant, whereas no significant difference was observed between NT-proBNP and cTnI measured with the Vcheck assay (Table 2). These findings suggest that the laboratory-based high-sensitivity cTnI assay may be more suitable for identifying dogs in very early stages of disease, before abnormalities become detectable by conventional screening methods.

## Discussion

This study evaluated the diagnostic performance of NT-proBNP and cTnI measured with the Vcheck V200 point-of-care assay in a large cohort of client-owned Doberman Pinschers. The study was not intended to re-establish NT-proBNP or cTnI as biomarkers of DCM, but to determine how these established biomarkers perform when measured with the Vcheck V200 point-of-care assays in this specific clinical population. A major strength of the study is the prospective longitudinal design, which enabled assessment of biomarker concentrations across different stages of disease progression. The study included 453 serum samples obtained from 345 dogs that underwent standardized clinical screening consisting of echocardiography, short ECG, and 24-h Holter monitoring. Furthermore, the inclusion of a unique “last normal” (LN) group allowed assessment of biomarker performance in dogs that were clinically classified as healthy at the time of sampling but developed DCM within the following year. Finally, the availability of paired Vcheck and IDEXX cTnI measurements enabled direct comparison between a point-of-care and a laboratory-based high-sensitivity assay.

Several important findings emerged from this study. Both NT-proBNP and cTnI measured with the Vcheck assay were significantly associated with DCM and provided clinically useful diagnostic information across different disease stages. Clinically applicable screening thresholds were identified for both biomarkers. However, cTnI measured with the laboratory-based IDEXX assay consistently achieved higher diagnostic accuracy than either Vcheck biomarker, particularly in dogs classified as LN. In addition, combining NT-proBNP and cTnI measurements increased diagnostic sensitivity but reduced specificity substantially.

NT-proBNP is a well-established biomarker of myocardial wall stress and has been widely used for screening of Doberman Pinschers at risk for DCM. Previous studies reported increased NT-proBNP concentrations in affected dogs and identified cutoff values ranging from approximately 550–626 pmol/L for detection of occult disease (11, 12). The cutoff value of 580 pmol/L identified in the present study is therefore in close agreement with previously published data and supports the reproducibility of NT-proBNP as a screening biomarker in this breed. Notably, this cutoff also corresponded to the ROC-derived optimal threshold based on the Youden index, further supporting its suitability for clinical screening applications.

In the present study, NT-proBNP showed its highest diagnostic performance in dogs with structural cardiac changes, particularly those classified as stage B2. This finding is consistent with the biological function of NT-proBNP, which is released in response to increased myocardial wall stress and ventricular remodeling (14). In contrast, diagnostic performance was lower in dogs with isolated ventricular arrhythmias (stage B1), supporting previous observations that NT-proBNP has limited ability to detect the arrhythmic phenotype of Doberman cardiomyopathy in the absence of echocardiographic abnormalities (11).

Cardiac troponin I is a sensitive and specific biomarker of myocardial injury and has previously been shown to be increased in Doberman Pinschers with DCM (15). In the present study, cTnI concentrations measured with both Vcheck and IDEXX increased with disease stage and were significantly higher in affected dogs than in healthy controls. The identified screening thresholds of 0.21 ng/mL for Vcheck and 0.11 ng/mL for IDEXX were highly comparable to previously reported cutoff values for conventional and high-sensitivity cTnI assays (15, 16), supporting the robustness of cTnI as a biomarker for DCM screening in Doberman Pinschers. In addition, these clinically selected thresholds were very similar to the ROC-derived optimal cutoff values identified in the present study, further supporting their applicability in clinical screening settings. Compared with NT-proBNP, cTnI reflects myocardial injury rather than hemodynamic stress. Consequently, increased cTnI concentrations may occur before measurable ventricular remodeling develops, potentially allowing detection of disease at an earlier stage. This concept is supported by previous studies using high-sensitivity cTnI assays in Doberman Pinschers (16) and is consistent with the findings of the present study.

A particular strength of the present study was the inclusion of dogs classified as LN. These dogs showed normal echocardiographic and Holter findings at the time of sample collection but developed DCM within the following year based on standard screening examinations. This group provides a unique opportunity to investigate biomarker alterations preceding conventional diagnostic abnormalities.

Among all biomarkers evaluated, cTnI measured with the IDEXX assay demonstrated the highest diagnostic accuracy in LN dogs. These findings support the hypothesis that subtle myocardial injury may be detectable before structural cardiac changes or clinically relevant arrhythmias become evident on routine screening examinations. The results are consistent with previous reports demonstrating improved early disease detection using high-sensitivity cTnI assays and identification of dogs that subsequently developed DCM despite initially normal screening results (12, 16) and further support the potential role of cTnI as an adjunctive screening tool for identifying Doberman Pinschers at increased risk of developing DCM.

Direct comparison of cTnI measurements obtained with the Vcheck and IDEXX assays revealed clear differences in diagnostic performance. Although both assays identified clinically useful screening thresholds and showed increasing concentrations with advancing disease stage, the IDEXX assay consistently achieved higher AUCs and greater specificity across all comparisons. This difference was particularly evident in LN dogs, in which IDEXX cTnI demonstrated substantially better discrimination between dogs that subsequently developed DCM and those that remained healthy.

The superior performance of the IDEXX assay is likely related to its higher analytical sensitivity. High-sensitivity cTnI assays are capable of detecting very small increases in circulating troponin concentrations and may therefore identify subtle myocardial injury before structural cardiac changes or clinically relevant arrhythmias become detectable. This interpretation is supported by previous findings in Doberman Pinschers demonstrating improved early disease detection with high-sensitivity cTnI assays (16). More generally, recent independent analytical validation of the Vcheck V200 NT-proBNP assay in cats has highlighted that point-of-care assay performance must be evaluated for the specific analyte, species, sample matrix, and intended clinical application. Although these feline data cannot be directly extrapolated to dogs or to cTnI measurement, they further emphasize that analytical performance and clinically applicable thresholds are assay- and species-specific (17).

Despite these differences, the Vcheck assay demonstrated clinically relevant diagnostic performance and offers practical advantages, including immediate availability of results and in-clinic testing. The feasibility of rapid point-of-care cTnI assessment is also supported by a recent study by Santarelli et al., who reported moderate to strong agreement between a qualitative rapid cTnI test and a quantitative laboratory assay in dogs and cats. However, because the investigated test format, analytical sensitivity, and intended clinical application differed substantially from those of the Vcheck assay, these findings should be interpreted as supporting the general concept of point-of-care troponin testing rather than as direct validation of the present assay (18). Therefore, when laboratory-based high-sensitivity cTnI measurement is unavailable or when rapid clinical decision-making is required, Vcheck may be used as an adjunctive point-of-care screening tool. However, the two assays should not be regarded as analytically interchangeable, because Vcheck yielded systematically higher cTnI concentrations and showed lower diagnostic accuracy, particularly in dogs with very early disease. Consequently, a normal Vcheck result does not reliably exclude DCM, and dogs with persistent clinical suspicion should still undergo comprehensive cardiac evaluation.

Combining NT-proBNP and cTnI measurements obtained with the Vcheck assay increased overall diagnostic sensitivity compared with either biomarker alone. More than 90% of dogs with DCM were correctly identified by the combined model. However, this improvement was accompanied by a substantial reduction in specificity, resulting in a higher number of false-positive classifications. This trade-off between sensitivity and specificity is expected when combining biomarkers that reflect different aspects of disease pathophysiology, namely myocardial wall stress and myocardial injury.

From a clinical perspective, the optimal screening strategy depends on the intended application. In breeding or screening programs, maximizing sensitivity may be desirable to minimize the risk of missing affected dogs, whereas higher specificity may be preferred when unnecessary follow-up examinations should be avoided. Similar findings were reported by Gordon et al., who demonstrated that combining physical examination variables with NT-proBNP improved discrimination between Doberman Pinschers without evidence of DCM and dogs with preclinical disease, although individual NT-proBNP and cTnI measurements also showed clinically useful diagnostic performance (19).

No relevant influence of age on NT-proBNP or cTnI concentrations was observed in the healthy control population. A small but statistically significant effect of sex was detected for NT-proBNP, with male dogs exhibiting slightly higher concentrations than females. Although the magnitude of this difference was limited and its clinical relevance remains uncertain, this finding may warrant further investigation in larger Doberman populations. In addition, no significant association between storage duration and measured biomarker concentrations was identified. However, this observational analysis does not establish analytical stability during prolonged frozen storage, and storage-related or other preanalytical effects cannot be excluded. Taken together, these findings support the clinical value of biomarker-based screening in Doberman Pinschers at risk for DCM. Although echocardiography and 24-h Holter monitoring remain the diagnostic gold standard, these examinations require specialized equipment and expertise that may not be readily available in all clinical settings. Biomarker testing therefore represents a practical adjunctive screening approach for identifying dogs that warrant comprehensive cardiac evaluation.

Among the evaluated biomarkers, cTnI measured with the IDEXX assay demonstrated the highest overall diagnostic accuracy, particularly for the detection of very early disease. However, both NT-proBNP and cTnI measured with the Vcheck assay provided clinically useful information and may assist veterinarians in identifying dogs at increased risk of DCM. Importantly, biomarker concentrations should not be interpreted in isolation but rather in conjunction with clinical findings and follow-up diagnostic testing. From a practical perspective, dogs exceeding the proposed screening thresholds should be considered candidates for referral for echocardiography and 24-h Holter monitoring. The integration of biomarker testing into routine health screening programs may facilitate earlier identification of affected dogs and support clinical decision-making in both primary care and breeding populations.

### Limitations

A limitation of the present study is that some dogs contributed multiple serum samples over time. Consequently, observations were not fully independent, and within-dog correlation was not accounted for using mixed-effects models, generalized estimating equations, or cluster-adjusted ROC analyses. This may have resulted in overly narrow confidence intervals and may have influenced *p*-values and estimates of diagnostic performance. At the same time, repeated examinations allowed evaluation of biomarker behavior across different stages of disease progression and reflected the longitudinal nature of DCM screening in Doberman Pinschers. The prolonged storage duration of some serum samples represents an additional limitation. Although no significant association between storage duration and measured biomarker concentrations was detected, the study did not include a prospective stability assessment. Therefore, storage-related matrix changes or other preanalytical effects cannot be excluded.

Another limitation is that some dogs with established DCM were receiving cardiac medication at the time of sample collection. Although treatment effects on biomarker concentrations were not specifically evaluated, medication may have influenced individual NT-proBNP and cTnI values.

Dogs with equivocal echocardiographic findings or uncertain stage assignment were excluded to ensure clear clinical classification. While this strengthened the reference standard used for diagnostic evaluation, it may have resulted in somewhat higher estimates of diagnostic accuracy than would be expected in a routine screening population that includes borderline cases.

Finally, some dogs classified as healthy may have been affected by very early disease that was not detectable by echocardiography or Holter monitoring at the time of examination. In addition, undetected subclinical extracardiac disease cannot be completely excluded despite clinical examination and routine diagnostic testing. Both factors may have contributed to overlap in biomarker concentrations between healthy and affected dogs. Renal function was not systematically assessed in all dogs. Although serum urea and creatinine concentrations were available in a limited number of dogs, these data were insufficient for a systematic evaluation of renal function. Therefore, mild or subclinical renal dysfunction and its potential influence on NT-proBNP concentrations cannot be completely excluded.

## Conclusion

This study demonstrates that the Vcheck V200 NT-proBNP and cTnI assays provide clinically useful diagnostic information for the detection of dilated cardiomyopathy in Doberman Pinschers. Clinically relevant screening thresholds were identified for NT-proBNP (580 pmol/L) and Vcheck cTnI (0.21 ng/mL), supporting the use of biomarker-based screening in dogs at risk for DCM.

Among the evaluated biomarkers, cTnI measured with the laboratory-based IDEXX assay showed the highest diagnostic accuracy, particularly for the detection of very early disease. Although the Vcheck assay did not achieve the same level of performance and should not be regarded as interchangeable with the IDEXX assay, it represents a practical in-clinic screening option when laboratory testing is unavailable or when an immediate result is required. Increased Vcheck biomarker concentrations should prompt comprehensive cardiac evaluation, including echocardiography and 24-h Holter monitoring, whereas a normal Vcheck result does not reliably exclude early DCM.

## Data Availability

The datasets supporting the conclusions of this article are available from the corresponding author upon reasonable request and subject to applicable institutional, ethical, and data protection requirements.
